# Phosphorylation of HSF1 at serine 326 residue is related to the maintenance of gynecologic cancer stem cells through expression of HSP27

**DOI:** 10.18632/oncotarget.16361

**Published:** 2017-03-18

**Authors:** Kazuyo Yasuda, Yoshihiko Hirohashi, Tasuku Mariya, Aiko Murai, Yuta Tabuchi, Takafumi Kuroda, Hiroki Kusumoto, Akari Takaya, Eri Yamamoto, Terufumi Kubo, Munehide Nakatsugawa, Takayuki Kanaseki, Tomohide Tsukahara, Yasuaki Tamura, Hiroshi Hirano, Tadashi Hasegawa, Tsuyoshi Saito, Noriyuki Sato, Toshihiko Torigoe

**Affiliations:** ^1^ Department of Pathology, Sapporo Medical University School of Medicine, Chuo-Ku, Sapporo 060-8556, Japan; ^2^ Department of Obstetrics and Gynecology, Sapporo Medical University School of Medicine, Chuo-Ku, Sapporo 060-8556, Japan; ^3^ Department of Surgical Pathology, Sapporo Medical University School of Medicine, Chuo-Ku, Sapporo 060-8556, Japan

**Keywords:** gynecological cancer, cancer stem cell, stress response, HSP27, HSF1

## Abstract

Cancer stem-like cells (CSCs)/ cancer-initiating cells (CICs) are defined by their higher tumor-initiating ability, self-renewal capacity and differentiation capacity. CSCs/CICs are resistant to several therapies including chemotherapy and radiotherapy. CSCs/CICs thus are thought to be responsible for recurrence and distant metastasis, and elucidation of the molecular mechanisms of CSCs/CICs are essential to design CSC/CIC-targeting therapy. In this study, we analyzed the molecular aspects of gynecological CSCs/CICs. Gynecological CSCs/CICs were isolated as ALDH1^high^ cell by Aldefluor assay. The gene expression profile of CSCs/CICs revealed that several genes related to stress responses are preferentially expressed in gynecological CSCs/CICs. Among the stress response genes, a small heat shock protein HSP27 has a role in the maintenance of gynecological CSCs/CICs. The upstream transcription factor of HSP27, heat shock factior-1 (HSF1) was activated by phosphorylation at serine 326 residue (pSer326) in CSCs/CICs, and phosphorylation at serine 326 residue is essential for induction of HSP27. Immunohistochemical staining using clinical ovarian cancer samples revealed that higher expressions of HSF1 pSer326 was related to poorer prognosis. These findings indicate that activation of HSF1 at Ser326 residue and transcription of HSP27 is related to the maintenance of gynecological CSCs/CICs.

## INTRODUCTION

In various organs, the histological architectures are systematized by a hierarchical differentiation model. Stem cells are located on the top of hierarchical differentiation model and are defined as subpopulation having self-renewal ability and capacity of differentiation into mature cells that can repopulate specific tissues and specific organs [1]. Likewise, cancer stem cells with similar characters of stem cells had long been hypothesized. The outline of cancer stem cells that we have referred to as cancer stem-like cells (CSCs)/ cancer-initiating cells (CICs) was proposed in the 1960′s [2, 3], and the framework of CSCs/CICs is thought to be very similar to that of normal stem cells. In 1994, John Dick and colleagues reported in their historical work that human acute myeloid leukemia is organized in a hierarchical differentiation model, and CD34^+^CD38^−^ cells are the stem cells of this model and only CD34^+^CD38^−^ cells can regenerate the disease [4]. In the following works, CSCs/CICs were also isolated from several solid malignancies including breast cancer, lung cancer, colon cancer and prostate cancer, and CSC model is well accepted in human malignancies [5]. CSCs/CICs are now defined as a small subpopulation of cancer cells that are endowed with ‘self renewal capacity’, ‘differentiation capacity’ and ‘higher tumorigenicity’ [2]. CSCs/CICs may contribute to tumor recurrence and metastasis by their resistance to chemotherapy-radiation therapy and cytotoxic chemotherapy [6]. Therefore eradication of CSCs/CICs is indispensable for radical cancer treatment.

A stress responsive system including heat shock proteins (HSPs) are protective mechanism for various types of stress and is conserved in that all cells are equipped with [7]. All living organisms on earth including humans are constantly exposed to stresses, such as active oxygen, warm temperature and low oxygen. Despite the severe environment, all organisms from sea-squirts to humans have the ability to survive because of the innate acquisition of a stress-responsive system. Normal stem cells are endowed with stress resistance to survive the stress and can regenerate the organ under stress conditions [8]. Recent studies revealed that CSCs/CICs like normal stem cells have distinctive stress-responsive systems compared with differentiated non-CSCs/CICs, and stress-responsive mechanisms are related to malignant phenotypes indicating the relation between stress response mechanisms and CSCs/CICs [9–14].

In present study, we performed transcriptome analysis of gynecological CSCs/CICs using a cDNA microarray, and found that a stress-responsive gene HSP27 is preferentially expressed in CSCs/CICs, that might be induced by constitutive activation of heat shock factor-1 (HSF1) by phosphorylation at serine 326 residue.

## RESULTS

### Isolation of CSCs/CICs from gynecological cancers

**Figure 1:**
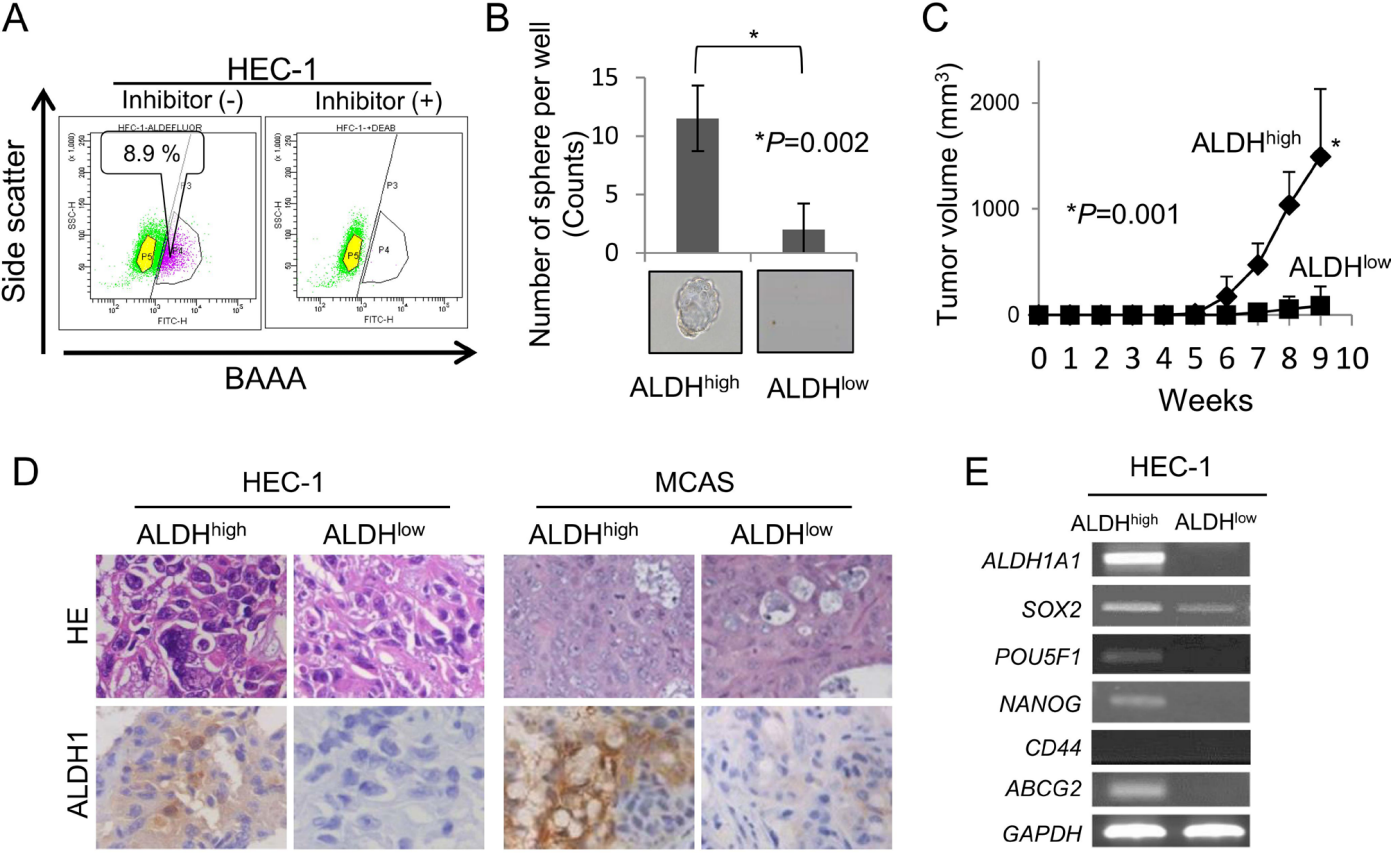
Isolation of CSCs/CICs from HEC-1 cells by Aldefluor assay (**A**) Detection of ALDH1high cells. ALDH1^high^ cells were isolated using HEC-1 cell. Percentage represents the proportion of ALDH1^high^ cells. (**B**) Representative picture of tumor sphere. ALDH1^high^ and ALDH1^low^ cells derived from HEC-1 cells were cultured in CSC Certified™ Complete Serum-Free Medium. After 2 weeks of culture *in vitro*, a picture of a tumor sphere was taken and sphere numbers were counted. Data represent means ± SD. (**C**) Tumor formation ability of HEC-1 ALDH1high and ALDH1low cells. ALDH1^high^ and ALDH1^low^ cells derived from HEC-1 cells were inoculated into the backs of NOD/SCID mice subcutaneously with serial dilution (10^2^ - 10^4^). Graphs show the tumor growth curves of ALDH1^high^ and ALDH1^low^ injected groups with injections of 10^4^ cells. Data represent means ± SD. Differences between ALDH1^high^ cells and ALDH1^low^ cells were examined for statistical significance using Student's *t-test*. *P value. (**D**) Histology of ALDH1high cell-derived and and ALDH1low cell-derived tumors. Tumors derived from ALDH1^high^ and ALDH1^low^ cells in HEC-1 and MCAS cells were stained by hematoxylin and eosin and immunostained by ALDH1 antibody. Magnification ×200. (**E**) Expressions of stem cell markers by RT-PCR analysis. ALDH1^high^ and ALDH1^low^ cells derived from HEC-1 cells were examined for expression of stem cell markers (SOX2, POU5F1, NANOG, CD44 and ABCG2). GAPDH was used as an internal control.

In the previous studies, we confirmed that CSCs/CICs were enriched in ALDH^high^ population from ovarian cancer line cells [15, 16]. To analyze the molecular aspects of CSCs/CICs in gynecologic cancers, we isolated ALDH^high^ cells from endometrioid adenocarcinoma line cell HEC-1 to isolate CSCs/CICs. The rate of ALDH^high^ cells was 8.9% by Aldefluor assay (Figure 1A). To confirm that CSCs/CICs were enriched in ALDH^high^ cells, a sphere forming assay, xenograft transplantation and RT-PCR analysis were performed. ALDH^high^ cells derived from HEC-1 cells showed significant higher sphere-formation in floating condition than that of ALDH^low^ cells (Figure 1B). To address the *in vivo* tumor-initiating ability of ALDH^high^, we injected 10^2^, 10^3^ and 10^4^ of ALDH^high^ cells and ALDH^low^ cells derived from HEC-1 cells into NOD/SCID mice. Tumor initiation was observed in 5 of 11 mice injected with 10^3^ of ALDH^high^ cells and 10 of 11 mice injected with 10^4^ of ALDH^high^ cells (Table 1). The estimated CSC/CIC frequency in ALDH^high^ cells was 1 in 3082 cells. On the other hand, tumor initiation was observed in 1 of 11 mice injected with 10^3^ of ALDH^low^ cells and 4 of 11 mice injected with 10^4^ of ALDH^low^ cells. The estimated CSC/CIC frequency in ALDH^low^ cells was 1 in 19987. No tumor initiation was observed in 10^2^ of ALDH^high^ cell and ALDH^low^ cell injections. The difference of estimated CSC/CIC frequency was statistically significant (*P* = 0.000258) (Table 1). The tumors derived from 10^4^ of ALDH1^high^ cells grew statistically significantly faster than those derived from ALDH^low^ cells (Figure 1C). Similar difference of tumor-initiation was observed in MCAS cells and HTBoA cells (Table 1). Tumors derived from ALDH^high^ and ALDH^low^ cells in HEC-1 and MCAS cells showed no notable histological difference. We then performed immunohistochemical staining using anti-ALDH1 antibody to determine the ALDH1 protein expression in tumors derived from ALDH^high^ and ALDH^low^ cells. The tumors derived from ALDH^high^ cells showed higher positivity to anti-ALDH1 antibody than that in the tumors derived from ALDH^low^ cells (Figure 1D). ALDH^high^ cells showed higher expressions of stem cell-related genes (ALDH1, SOX2, POU5F1 and NANOG) at higher levels than did ALDH^low^ cells (Figure 1E). These results indicate that ALDH^high^ cells derived from HEC-1 cells are enriched with CSCs/CICs. In our previous study, we showed that ALDH^high^ cells from ovarian cancer line cells MCAS and HTBoA were also enriched with CSCs/CICs [16, 17]. We therefore further analyzed ALDH^high^ cells derived from HEC-1, MCAS and HTBoA cells to address the molecular insight of gynecological CSCs/CICs.

**Table 1:** Tumor-initiating ability of endometrial cancer cell and ovarian cancer cells

	Cells	Injected cell number					
		10^2^	10^3^	10^4^	CSC frequency	95% CI	*P* value
HEC-1	ALDH^high^cells	0/11	5/11	10/11	1 in 3082	1546–6145	*0.000258
	ALDH^low^cells	0/11	1/11	4/11	1 in 19987	8195–48748	
	control siRNA	-	3/5	-			
	HSF1 siRNA-i	-	0/5	-			
	HSF1 siRNA-ii	-	0/5	-			
MCAS	ALDH^high^cells	0/5	0/5	5/5	1 in 4326	1640–11415	0.0519
	ALDH^low^cells	0/5	0/5	2/5	1 in 22379	5622–89084	
HTBoA	ALDH^high^cells	0/5	0/5	3/5	1 in 12859	4134–39996	*0.0214
	ALDH^low^cells	0/5	0/5	0/5	-	18527-	

The tumor-initiating abilities were evaluatad at day 70 post cell injection. Data are expressed as number of tumors formed/number of injections. Differences of estimated frequencies of CICs were analyzed by Chi-square test. CSC frequencies were calclutated at ELDA web site (http://bioinf.wehi.edu.au/software/elda/). *P < 0.05.

### Stress responsive genes are expressed in ALDH^high^ cells

**Figure 2:**
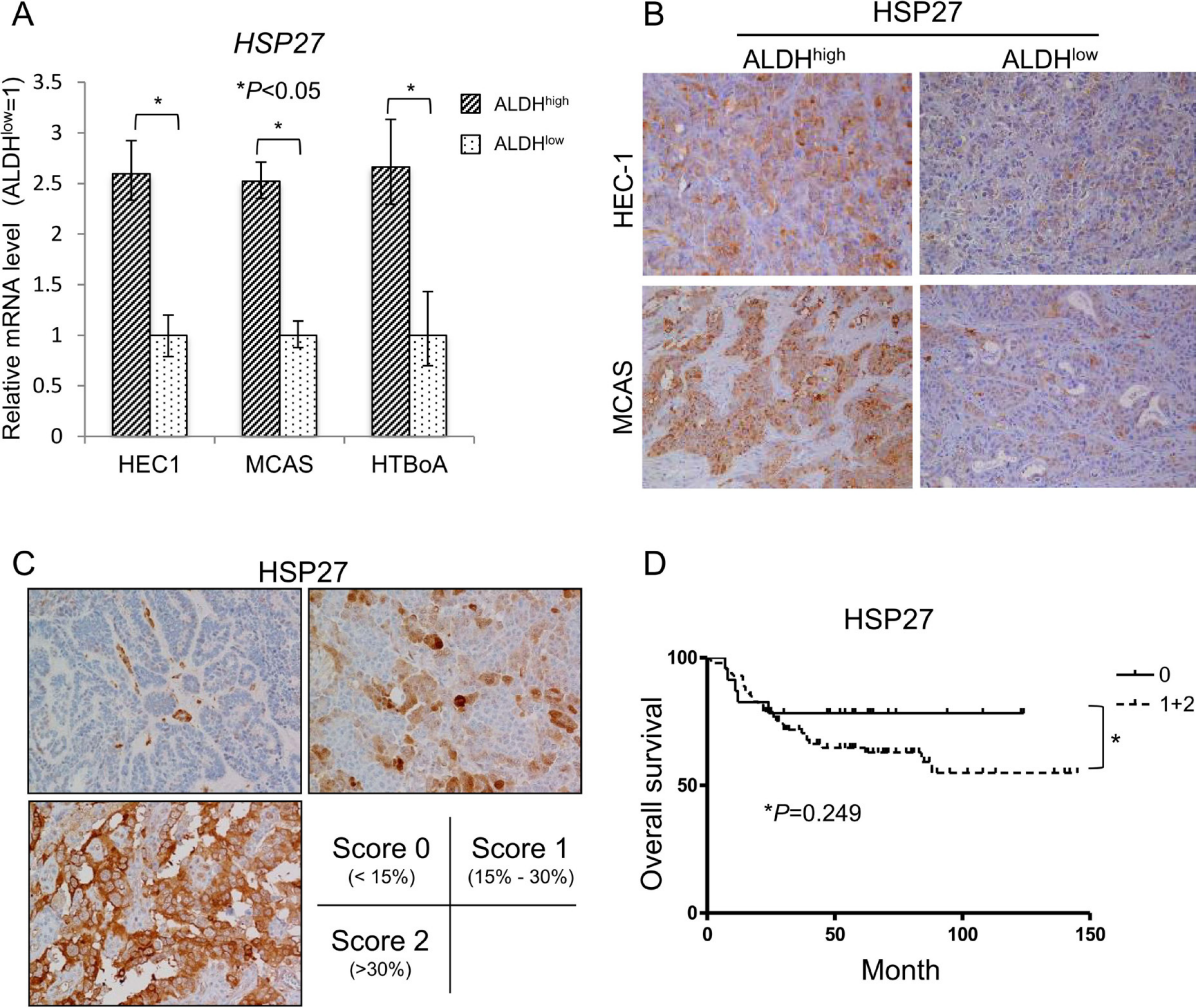
HSP27 expressed in ALDH1^high^ cells derived from endometrial and ovarian carcinoma (**A**) HSP27 expression by qRT-PCR. HSP27 expression was assessed by real-time PCR using ALDH1^high^ and ALDH1^low^ cells derived from HEC-1, MCAS and HTBoA cells. Data represent means ± SD. *P values. (**B**) The expression of HSP27 in tumors derived from ALDHhigh cells and ALDHlow cell. Tumors derived from ALDH1^high^ cells and ALDH1^low^ cells in HEC-1 and MCAS cells were immunohistochemically stained by HSP27 antibody. Magnification ×200. (**C**) The expression of HSP27 in clinical samples. HSP27 protein expression was addressed using anti-HSP27 antibody. Total of 122 ovarian cancer cases were immunohistochemically stained. The expressions of HSP27 were evaluated as (score 0: < 15%, *n* = 40; score 1: 15%–30%, *n* = 41; score 2: > 30%, *n* = 41). (**D**) Overall survival. The differences of overall survival were examined for statistical significance using Fischer's test. *P values.

To analyze the molecular mechanisms of ALDH^high^ cells, we screened ALDH^high^ cell-specific genes using a cDNA microarray. The summary of up-regulated genes in ALDH^high^ cells derived from HEC-1 cells is shown in Supplementary Table 2. Interestingly, several genes involved in stress response are expressed in ALDH^high^ cell-specific expression. The expressions of HSP27 mRNA in ALDH^high^ and ALDH^low^ cells derived from HEC1, MCAS and HTBoA were confirmed by qRT-PCR (Figure 2A). HSP27 protein expressions in the tumors derived from ALDH^high^ cells were examined by immunohistochemical staining using anti-HSP27 antibody. The tumors derived from ALDH^high^ cells derived from HEC-1 cells and MCAS cells showed higher expressions of HSP27 protein than those in tumors derived from ALDH^low^ cells (Figure 2B). To confirm HSP27 protein expressions in clinical samples, immunohistochemical staining using human ovarian cancer specimens (*n* = 122) were performed. Ovarian cancer cases showed positive staining for HSP27, and we categorized the cases into 3 groups (score 0: < 15%, *n* = 40; score 1: 15% - 30%, *n* = 41; score 2: > 30%, *n* = 41) according to the positivity for HSP27 staining (Figure 2C). Lower HSP27 staining (score 0) showed relative better prognosis than that with higher HSP27 staining (score 1 + score 2); however, the difference did not reach statistical significance (*P* = 0.249) (Figure 2D).

### HSP27, a heat shock protein family has a role in the maintenance of CSCs/CICs

**Figure 3:**
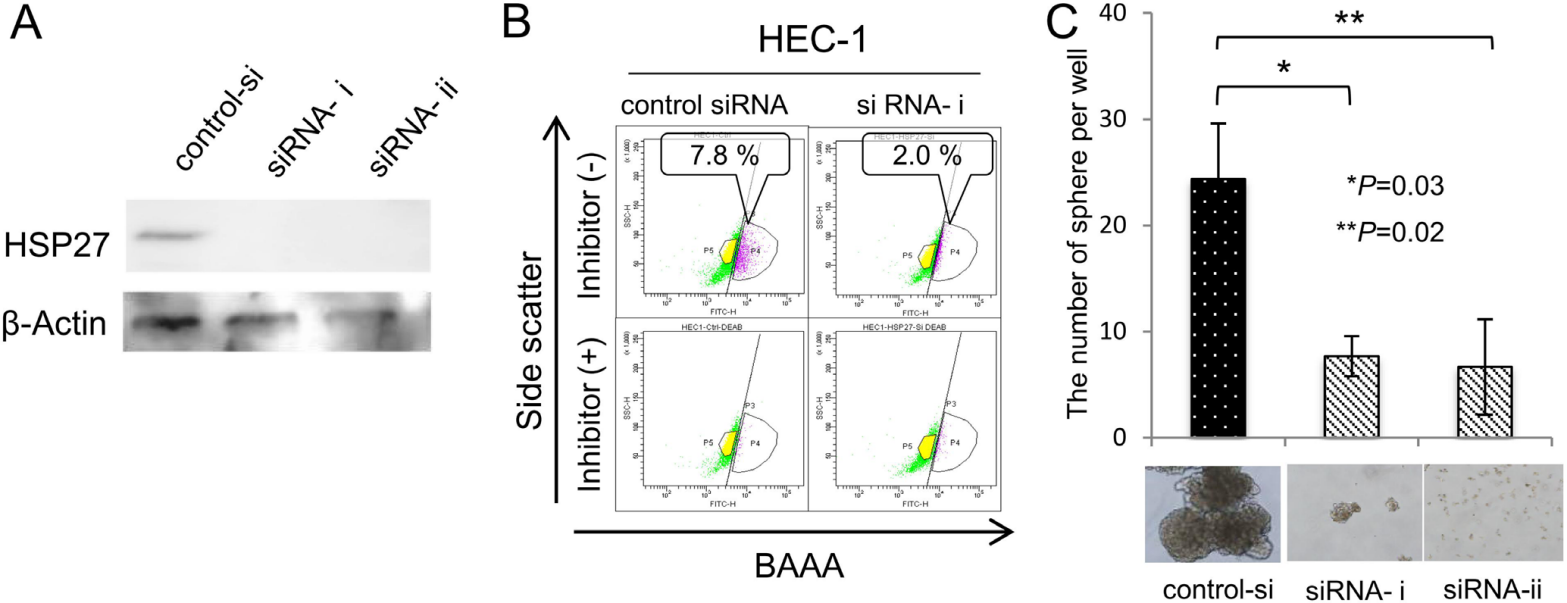
HSP27 has a role in the maintenance of CSCs/CICs (**A**) Western blotting of HSP27 knockdown cells. HSP27 siRNA was transfected into HEC-1 cells. Twenty-four hours after transfection, total RNAs were purified and the expression of HSP27 was evaluated by Western blotting. β-actin was used as an internal control. (**B**) Aldefluor assay. ALDH activity of HSP27 knockdown cells was detected using the Aldefluor assay 48 hours after transfection. Percentage represents the proportion of ALDH1^high^ cells. (**C**) Sphere formation assay. ALDH1^high^ and ALDH1^low^ cells derived from HSP27 SiRNA-transfected HEC1 cells were cultured in serum-free medium. After 2 weeks of culture *in vitro*, a picture of a tumor sphere was taken. The counts were examined for statistical significance using Student's *t-test*. Data represent means ± SD. *P values.

A previous study described that HSP27 has a role in the resistance to apoptosis induced by hypoxia or serum depletion in CD133^+^ colon cancer-initiating cells [18]. And, other study revealed that HSP27 protein expression is related to poorer prognosis in glioma cases [19]. We therefore focused on HSP27, and analyzed its function in gynecologic CSCs/CICs. HSP27 protein downregulation by HSP27-specific siRNAs were confirmed by a Western blot (Figure 3A). HSP27 protein knockdown reduced the frequency of ALDH^high^ cells and sphere-forming ability compared with control siRNA transfected HEC-1 cells (Figure 3B and 3C). These results indicate that HSP27 has a role in the maintenance of CSCs/CICs.

### Stress-activated transcription factor HSF1 works upstream of HSP27

**Figure 4:**
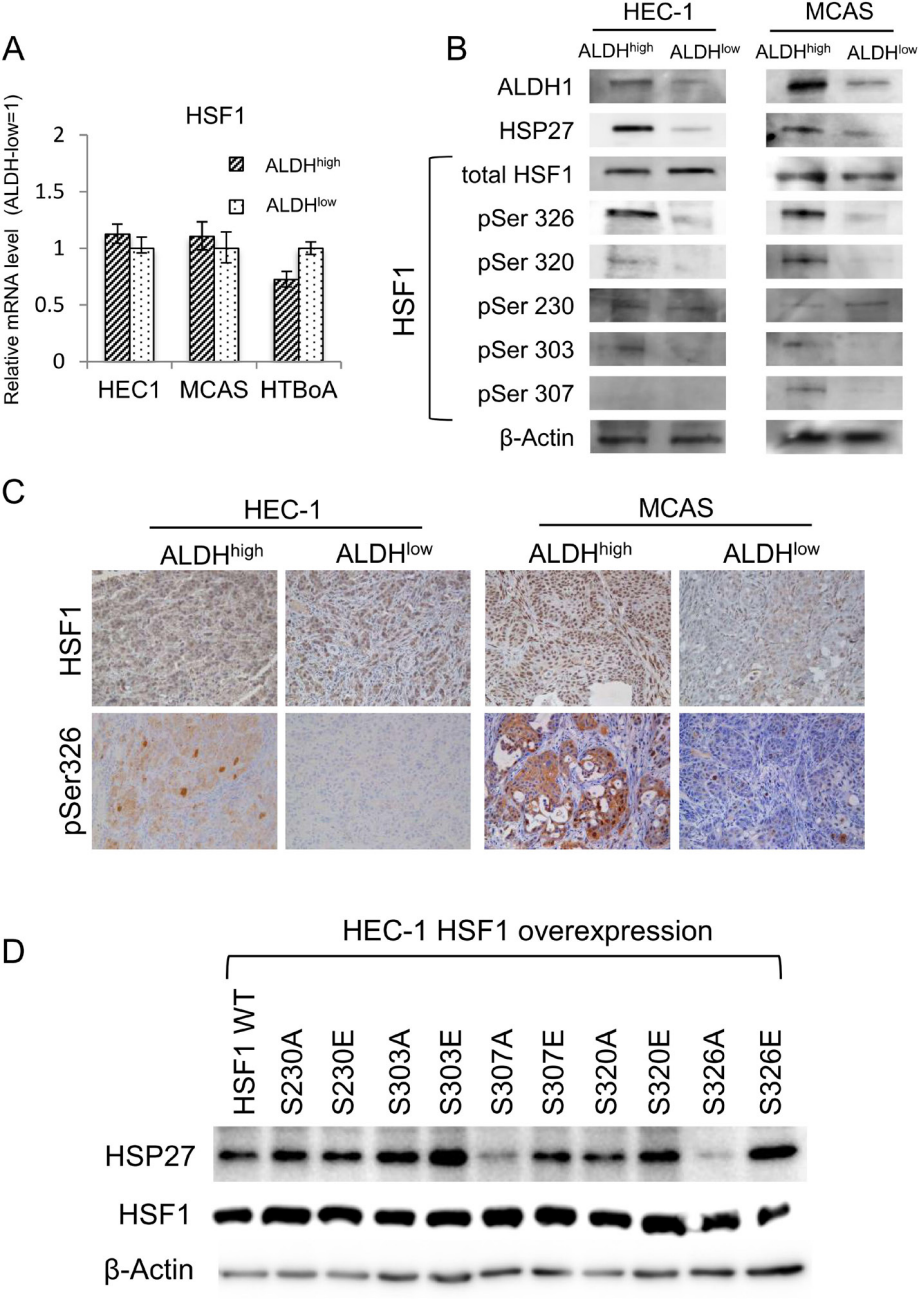
Stress-activated transcription factor HSF1 works upstream of HSP27 (**A**) HSF1 expression by qRT-PCR analysis. HSF1 expression was assessed by qRT-PCR using ALDH1^high^ and ALDH1^low^ cells derived from HEC-1, MCAS and HTBoA cells. GAPDH was used as an internal control. (**B**) Western blotting analysis. ALDH1^high^ cells and ALDH1^low^ cells in HEC-1 and MCAS cells were used. The expression of ALDH1, HSP27, HSF1 and phospho-HSF1 (pSer326, pSer320, pSer230, pSer303 and pSer307) was evaluated by Western blotting. β-actin was used as an internal control. (**C**) Immunohistochemical findings of Ser326-phosphorylated HSF1. Tumors derived from ALDH1^high^ cells and ALDH1^low^ cells of HEC-1 and MCAS cells were immunohistochemically stained using anti-phospho-HSF1 (pSer326) antibody and anti-HSF1 antibody. Magnification ×200. (**D**) Expressions of HSP27 in HSF1 mutants overexpressed HEC-1 cells. HEC-1 cells were overexpressed with HSF1 wild type (WT) and mutants (S230A, S230E, S303A, S303E, S307A, S307E, S320A, S320E, S326A and S326E). The expression of HSP27 protein and total HSF1 were analyzed by Western blots. β-actin was used as an internal control.

Since the expression of HSP27 is positively regulated by stress responsive transcription factor HSF1, we thus hypothesized that HFS1 is activated in CSCs/CICs [20]. The expression of HSF1 mRNA was addressed by qRT-PCR, and there were no differences between expression levels in ALDH^high^ cells and ALDH^low^ cells (Figure 4A). The protein expression levels of HSF1 in ALDH^high^ cells an ALDH^low^ cells did not show significant differences (Figure 4B). HSF1 is phosphorylated at numbers of serine residues during cellular stress, and phosphorylation at Ser230 and Ser326 are essential for activation of HSF1 [21, 22]. We thus addressed the levels of phosphor HSF1 levels using phosho-HSF1 specific antibodies (pSer326, pSer320, pSer230, pSer303 and pSer307). HSF1 pSer326 were significantly increased in ALDH^high^ cells than that in ALDH^low^ cells derived from both HEC-1 cells and MCAS cells; however, other phosphorylation sites including Ser230 did not show any differences (Figure 4B). Immunohistochemical staining revealed that tumors derived from ALDH^high^ cells show higher phosphorylation at Ser326, whereas the protein expression levels of HSF1 did not show any difference in tumors derived from ALDH^high^ cells and ALDH^low^ cells (Figure 4C). To confirm the function of HSF1 pSer326, several HSF1 mutants were constructed. Ser230, Ser303, Ser307, Ser320 and Ser326 residues were substituted to alanine (A) or glutamic acid (E) to mimic non-phosphorylated status and phosphorylated status, respectively. HEC-1 cells were transfected with HSF1 mutants and stable transformants were established. The total HSF1 expression levels were confirmed by a Western blot (Figure 4D). HSP27 protein expression was increased by S303E, S320E and S326E overexpression. On the other hand, HSP27 protein expression was decreased by S307A and S326A overexpression. These observations indicate that phosphorylation of HSF1 at serine 326 residue is essential in the transcription of HSP27, and phosphorylation at serine 303 and 320 residues might have roles in the transcription of HSP27.

### Phosphorylation of HSF1 at Ser326 is related to the maintenance of CSCs/CICs and poorer prognosis

**Figure 5:**
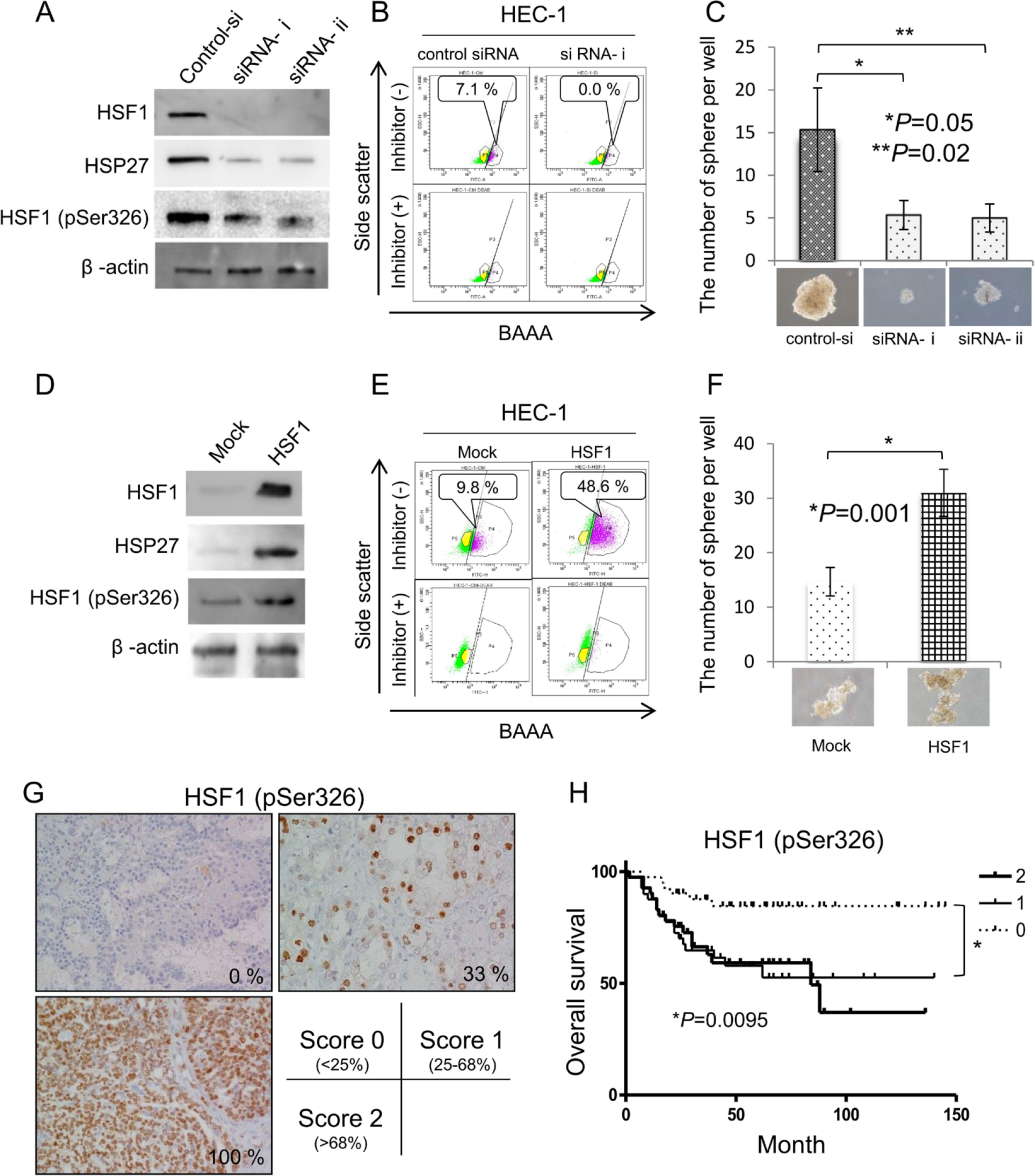
Phosphorylation of HSF1 at Ser326 is related to the maintenance of CSCs/CICs and poorer prognosis (**A**) Western blotting of HSF1 knockdown cells. HSF1 siRNA was transfected into HEC-1 cells. Twenty-four hours after transfection, total RNAs were purified and the expression of HSF1, HSF1 pSer326 and HSP27 were evaluated by Western blotting. β-actin was used as an internal control. (**B**) Aldefluor assay. ALDH activity of HSF1 knockdown cells was detected using the Aldefluor assay 48 hours after transfection. Percentage represents the proportion of ALDH1^high^ cells. (**C**) Sphere formation assay. ALDH1^high^ and ALDH1^low^ cells derived from HSF1 siRNA-transfected HEC1 cells were cultured in serum-free medium. After 2 weeks of culture *in vitro*, a picture of a tumor sphere was taken. The counts were examined for statistical significance using Student's *t-test*. Data represent means ± SD. *P values. (**D**) Western blotting of HSF1-overexpressed cells. Cells stably transfected with HSF1 and control vector-transfected cells were used. The expression of HSF1, HSF1 pSer326 and HSP27 were evaluated by Western blotting. β-actin was used as an internal control. (**E**) Aldefluor assay. ALDH activity of HSF1-overexpressed cells was detected using the Aldefluor assay 48 hours after transfection. Percentage represents the proportion of ALDH1^high^ cells. (**F**) Sphere formation assay. ALDH1^high^ and ALDH1^low^ cells derived from HSF1-overexpressed HEC1 cells were cultured in serum-free medium. After 2 weeks of culture *in vitro*, a picture of a tumor sphere was taken. The counts were examined for statistical significance using Student's *t-test*. Data represent means ± SD. *P values. (**G**) Immunohistological staining of phosphoHSF1 (pSer326). A total 122 of epithelial ovarian cancer tissues were immunohistochemically stained with anti-phopho-HSF1 pSer326 antibody and the expressions of HSF1 pSer326 were evaluated as (score 0: < 25%, *n* = 41; score 1: 25%–68%, *n* = 40; score 2: > 68%, *n* = 41). Representative pictures are shown. The HSF1 pSer326 positive rates are 0%, 33% and 100%, respectively. Magnification ×200. (**H**) HSF1 pSer326 positive cases are associated with poor prognosis. The differences of overall survival were examined for statistical significance using Fischer's test. *P values.

To address the functions of HSF1, we performed gene knockdown and overexpression of HSF1. HSF1 was specifically knocked down using 2 different HSF1 specific siRNAs and knockdown was confirmed by Western blot (Figure 5A). HSF1 pSer326 and HSP27 were also decreased by HSF1 knockdown (Figure 5A). The rates of ALDH^high^ cells and sphere-forming ability were decreased by HSF1 knockdown (Figure 5B and 5C). On the other hand, the levels of HSF1 pSer326 and HSP27, the rates of ALDH^high^ cells and sphere-forming ability were increased by HSF1 gene overexpression (Figure 5D–5F). These observations indicate that HSF1 pSer326 is related to the expression of HSP27 and is also related to the maintenance of CSCs/CICs. To further analysis the functions of HSF1 pSer326, we examined the CSC/CIC frequencies of HEC-1 cell overexpressed HSF1 mutants by limiting dilution assay. Stable transformants of HEC-1 cells with S303A, S303E, S320A, S320E, S326A and S326E mutant were addressed, and only S326E overexpressed HEC-1 cells showed statistically significant increase CSC/CIC frequency compared with negative control (Table 2). Overexpression of S326A showed tendency of decrease CSC/CIC frequency (Table 2). These results indicate that HSF1 phosphorylation at serine 326 is essential for the maintenance of CSCs/CICs.

**Table 2:** Stem cell frequency of HSF1 mutant overexpressed HEC1 cells

Cells	sphere-positive wells/total wells				CSC frequency	95% CI	^†^*P* value
	1 cell/well	10 cells /well	100 cells/well	1000 cells /well			
S303A	0/48	2/48	21/48	48/48	1 in 175	121.3–252	0.64
S303E	0/48	3/48	16/48	48/48	1 in 212	148.3–304	0.78
S320A	0/48	7/48	18/48	48/48	1 in 168	116.3–242	0.524
S320E	0/48	4/48	21/48	48/48	1 in 163	112.7–235	0.45
S326A	2/48	4/48	10/48	48/48	1 in 246	174.2–349	0.382
S326E	3/48	5/48	25/48	48/48	1 in 118	82.9–167	0.039*
WT	0/48	6/48	27/48	46/48	1 in 173	119.7–249	0.576
Mock	0/48	5/48	20/48	47/48	1 in 197	137.4–284	

^†^Defference with Mock cells were caliculated by Chi-square test. *P < 0.05.

To address the clinical significance of HSF1 pSer326, a total 122 of epithelial ovarian cancer tissues were immunohistochemically stained with anti-phosphoHSF1 (pSer326) rabbit polyclonal antibody (patients clinicopathological status is summarized in Supplementary Table 3). We divided into three groups (Figure 5G): Score 0 (HSF1 pSer326 positivity; < 25%, *n* = 41), Score 1 (HSF1 pSre326 positivity; 25%–68%, *n* = 40) and Score 2 (HSF1 pSer326 positivity; > 68%, *n* = 41). As summarized in Supplementary Table 3, there was no significant correlation between expression level of pHSF1, and age. FIGO clinical stage was significantly correlated with HSF1 (pSer326) expression. The univariate analysis revealed that HSF1 pSer326 score 1 or score 2, advanced FIGO stage, platinum resistant and optimal debulking surgery are significantly correlated with poorer prognosis (Table 3). However, only platinum resistant and optimal debulking surgery are correlated with poorer prognosis by multivariate analysis. Log-rank test revealed that higher expression of HSF1 pSer326 (Score1 and Score 2) are associated with poorer prognosis with a significant difference than those of lower expression levels of HSF1 pSer326 (*P* = 0.0095) (Figure 5H). Higher expression levels of phosphor-HSF1 (pSer326) showed tendency with shorter overall survival (OS) than those of lower expression levels.

**Table 3:** Summary of univariate analysis and multivariate analysis of overall survival

Factor	Total cases (*n* = 122)			
	Univariate analysis		Multivariate analysis	
	Risk ratio label (95% confidence interval)	*P*	Risk ratio label (95% confidence interval)	*P*
HSF1(pSer326) expression (Score 0~+2)				
0	1	-		
1	3.56 (1.40–9.04)	0.008**		
2	3.57 (1.41–9.08)	0.007**		
Advanced age (over 50)	1.78 (0.82–3.87)	0.14		
Multipara (≧2)	1.78 (0.70–3.24)	0.12		
Histological subtype				
Serous	1.55 (0.82–2.92)	0.17		
Cleacell	0.81 (0.40–1.63)	0.55		
Endometrioid	0.50 (0.16–1.64)	0.26		
Mucinous	0.97 (0.23–4.02)	0.97		
Advanced Stage (over FIGO Stage III)	8.02 (2.85–22.59)	< 0.001**		
Peritoneal dissemination	4.73 (1.93–9.90)	< 0.001**		
Lymph node metastasis	1.48 (0.78–2.81)	0.23		
Platinum resistant	12.46 (5.18–29.96)	< 0.001**	7.35 (2.72–19.87)	< 0.001**
Optimal debulking surgery	0.14 (0.07–0.30)	< 0.001**	0.40 (0.17–0.94)	0.04*

## DISCUSSION

Cancer stem-like cells (CSCs)/ cancer-initiating cells (CICs) are resistant to anticancer drugs and radiotherapy and they can survive therapies. Therefore, we suspect that CSCs/CICs have a high stress tolerance compared to that of non-CSCs/CICs. In this study, we investigated the molecular mechanism of the gynecological CSCs/CICs isolated as ALDH^high^ cells, and found CSCs/CICs have distinctive stress response mechanisms. Identification of rare subpopulations of cancer stem cells has created a new focus in cancer research in recent years, and gynecological CSCs/CICs have successfully isolated by using several markers; cell surface antigens [23, 24], and side population (SP) cells [25, 26] and Aldefluor assay [15, 27]. Overlapping subpopulation of ALDH^high^ and SP cells showed higher tumor-initiating ability compared with ALDH^high^ cells and SP cells indicating that those markers of CSCs/CICs are not definite CSC/CIC marker [16].

ALDH1 belongs to the ALDH family of enzymes that metabolize aldehydes to carboxylic acids. Aldehyde dehydrogenases (ALDHs) oxidize aldehydes to the corresponding carboxylic acids using either NAD or NADP as a coenzyme. Aldehydes are highly reactive aliphatic or aromatic molecules that play an important role in numerous physiological, pathological, and pharmacological processes. ALDH1 has been found in practically all organisms, and there are multiple isoforms with multiple subcellular localizations [28]. The ALDH family consists of 25 iso-enzymes, and ALDH1 can convert retinol to retinoic acid [28]. ALDH1 thus had been suggested to play a role in the expression of normal tissue stem cells through oxidation of retinol to control stem cell differentiation [29]. ALDH1 expression is increased in hematopoietic, neuronal, mesenchymal, endothelial and progenitor cells [29–31]. ALDHs have roles in anti-stress responses including oxidative stress and UV radiation, and now ALDH high active populations (ALDH^high^ cells) were well accepted as isolation of CSCs/CICs [32]. In this study, we used Aldefluor assay for isolation of ALDH^high^ cells from gynecological cancers. ALDH^high^ cells showed higher tumor-initiating ability, sphere-forming ability and higher expressions of stem cell-related genes, indicating that ALDH^high^ cells are enriched with CSCs/CISs as previous reports described. Therefore, ALDH^high^ cells used in this study are reasonable source for analysis of CSCs/CICs.

In this study, we screened the genes expressed in CSCs/CICs by using a cDNA microarray. And we found that several genes related to stress responses are overexpressed in CSCs/CICs. In a previous study, we showed that stress responsive signal transduction kinase MAPK13 is overexpressed in CSCs/CICs [14]. Gene knock down of MAPK13 significantly decreased tumor-initiation ability and sphere-forming ability, thus MAPK13 has a role in the maintenance of CSCs/CICs. In this study, we further focused on small heat shock protein family HSP27. Several HSP families have been described to tumor development, and HSP27 is also the case [33]. A recent study reported that breast CSCs/CICs isolated as ALDH^high^ cells or CD44^+^CD24^−^ cells showed higher HSP27 expressions and higher expressions of phosphorylated HSP27 [34]. They found that epithelial-mesenchymal transition (EMT) and activation of NF-κB signaling were related to HSP27 expression. The phenotypes of CSCs/CICs could be inhibited by an HSP27 inhibitor Qercetin or HSP27 specific gene knockdown using siRNAs. The other study identified HSP27 is overexpressed in CD133^+^ putative CSCs/CICs induced by hypoxia culture of colon cancer cells HT-29 [18]. They found that p38MAPK-MAPKAPK2 signaling has a role in the activation of HSP27. In this and our previous study, we also identified that both MAPK13 and HSP27 are preferentially expressed in CSCs/CICs. MAPK13-HSP27 signaling might also be activated in gynecological CSCs/CICs. Furthermore, a proteome analysis revealed that histological high grade of glioma cases were related to higher expressions of HSP27 protein [19]. In this study, we found that HSP27 is preferentially expressed in gynecological CSCs/CICs for the first time. HSP27 specific gene knockdown decreased the frequencies of ALDH^high^ cells and decreased the sphere-forming ability. These findings indicate that HSP27 has essential role in the maintenance of gynecological CSCs/CICs. The exact molecular mechanisms how HSP27 maintains gynecological CSCs/CICs are still elusive; however, targeting HSP27 might be a novel approach to target treatment-resistant CSCs/CICs and Quercetin is a possible candidate to target HSP27 [34]. A recent study revealed that HSP27 can be a target of cytotoxic T lymphocytes (CTLs), and HSP27-specific CTLs could recognize HSP27-positive myeloma cells [35]. Since chemotherapy-resistant CSCs/CICs are susceptible to CTLs [36], CSCs/CICs-targeting immunotherapy using HSP27 might be feasible. And, our recent findings indicate the potencies of CSC/CIC-targeting immunotherapy [10, 37–39]. However, HSP27 is also expressed in other normal organs and immunological responses to HSP27 may cause autoimmune diseases [33].

HSP27 expression is induced by activation of a transcription factor HSF1. In activation of HSF1, it undergoes trimerization and is localized to nucleus. HSF1 is phosphorylated at several residues under heat shock [40]. In normal cells, HSF1 regulates the various balance and homeostasis of cells. HSF1 and HSF2 play roles in spermatogenesis [41] and maintenance of the olfactory epithelium [42]. Recent studies described that HSF1 is also associated with carcinogenesis and poor prognosis [33, 43, 44] In this study, we showed that HSF1 down-regulation and HSF1 overexpression affect sphere-formation ability under non-stress conditions. These results indicate that HSF1 has a role in the maintenance of CSCs/CICs regardless of stress. Human HSF1 is phosphorylated on transactivation sites and transrepression sites [22]. Our results showed that the HSF1 protein was phosphorylated at Ser326 residue in ALDH1^high^ cells and has a role in transcription of HSP27 and maintenance of CSCs/CICs. IHC staining showed that high expression of HSF1 pSer326 is related to poorer prognosis by univariate analysis. However, multivariate analysis revealed that only Platinum resistant and Optimal debulking surgery are related to poorer prognosis. Since activation of HSF1 is related to stress-resistance including resistance to platinum, platinum resistance might diminished the phosphorylated HSF1 as an independent prognostic factor.

A recent study demonstrated that the kinase mTOR has a role in regulating the stress response and HSF1 phosphorylation on Ser326 [45]. And PI3K/AKT/mTOR signaling is activated in CSCs/CICs and mTOR is thought to be a molecular target of CSC/CIC-targeting therapy [46]. Thus, activated mTOR might phosphorylate HSF1 at Ser326 residue and induce the expression of HSP27 in CSCs/CICs, and mTOR, HSF1 and HSP27 might be candidates for CSC/CIC-targeting therapy.

In summary, we identified several stress-responsive genes are up-regurated in gynecological CSCs/CICs. Among stress-responsive gene products, HSP27 has a role in the maintenance of CSCs/CICs. HSF1 a transcription factor of HSP27 is activated in CSCs/CICs by phosphorylation at Ser326 residue. These results indicate that CSCs/CICs have distinctive stress-response mechanisms that may related to treatment resistance. HSF1 and HSP27 might be novel targets to target treatment resistant CSCs/CICs.

## MATERIALS AND METHODS

### Ethics statement

Mice were maintained and experimented on in accordance with the guidelines of and after approval by the Committee of Sapporo Medical University School of Medicine, Animal Experimentation Center under permit number 08-006. Any animal found unhealthy or sick was promptly euthanized. All studies were approved by the Institutional Review Board (IRB) of Sapporo Medical University Hospital. Written informed consent was obtained from all patients according to the guidelines of the Declaration of Helsinki.

### Cell lines and cell culture

Human endometrial carcinoma cells (HEC-1 cells) and human ovarian cells (MCAS and HTBoA cells) were obtained from ATCC (Manassas, VA, USA). HEC-1 and MCAS cells were maintained in Minimum Essential medium (MEM) (Life Technologies, Grand Island, NY, USA). HTBoA cells were maintained in Dulbecco's modified Eagle's medium (DMEM) (Sigma-Aldrich, St Louis, MO, USA). Each cell line was supplemented with 10% FBS and cultured in a humidified 5% CO2 incubator at 37°C.

### Aldefluor assay

The Aldefluor assay (Stem Cell Technologies™, Vancouver, BC, Canada) was performed to determine ALDH^high^ cells as described previously [15, 16]. Briefly, cells were counted and suspended in assay buffer containing 1 μM per 1 × 10^6^ cells of the ALDH substrate, boron- dipyrrometheneaminoacetaldehyde (BAAA), and incubated for 50min at 37°C. Each sample was treated with 50 nM of an ALDH-specific inhibitor, diethylaminobenzalydehyde (DEAB), as a negative control. BAAA-stained cells were analyzed and sorted using BD FACSAria™ II (BD Biosciences, San Jose, CA, USA).

### Sphere formation assay

Sphere forming assay using ALDH^high^ cells, ALDH^low^ cells, HSP27 siRNA transfected cells, HSF1 siRNA transfected cells and HSF1 plasmid transfected cells were performed as described previously [17]. Briefly, a total of 1,000 cells derived from HEC-1 cells were incubated in CSC Certified™ Complete Serum-Free Medium (Cell Systems Corporation, Kirkland, WA) in an Ultra-Low Attachment Surface culture 6-well plate (Corning^®^), and the number of spheres over 50 μm in diameter was counted under light microscopy.

For estimation of CSC/CIC frequencies, limiting dilution analysis was performed. Serially diluted HSF1 wild type or mutant (S303A, S303E, S320A, S320E, S326A and S326E) overexpressed HEC-1 cells were seeded into 96-well Ultra-Low Attachment Plate (Corning^®^) in sphere-forming medium for 14 days. Then sphere-forming wells were counted and estimated frequencies of CSC/CIC were calculated at ELDA web site (http://bioinf.wehi.edu.au/software/elda/) [47].

### Xenograft transplantation

Sorted cells were collected and re-suspended at the concentrations of 10^2^–10^4^ cells per 50 μl of PBS and mixed with 50 μl of matrigel (BD Biosciences). The cell-matrigel mixture was subcutaneously injected in the subcutaneous spaces of 6-week-old non-obese diabetic/severe combined immune - deficiency (NOD/SCID) mice (NOD.CB17-Prdkcscid/J, Charles River Laboratory, Yokohama, Japan) under anesthesia. Tumor growth was monitored weekly, and tumor volume was calculated by XY^2^/2 (X = long axis, Y = short axis). For the estimation of frequencies of CSCs/CICs ELDA web site (http://bioinf.wehi.edu.au/software/elda/) was used [47].

### Gene expression profiling using cDNA microarrays

RNAs from ALDH^high^ cells were labeled with Cy5 dye and those from ALDH^low^ cells were labeled with Cy3 dye and cDNA microarray using Human Panorama Micro Array (Sigma-Aldrich) was performed as described previously [48]. A dye-swap experiment (labeling ALDH^high^ cells and ALDH^low^ cells with Cy3 and Cy5, respectively) was also performed. Microarray raw data have been deposited in the Array Express data-base (E-MTAB-4440).

### Reverse transcription-polymerase analysis (RT-PCR) analysis and Quantitative real-time RT-PCR analysis (qRT-PCR)

Isolation of RNA and RT-PCR analysis were performed as described previously [49]. The thermal cycling conditions were 94°C for 2 min, followed by 35 cycles of 15 sec at 94°C, 30 sec at 60°C, and 30 sec at 72°C. GAPDH was used as an internal positive control. The primers used in experiments are summarized in Supplementary Table 1.

Quantitative real-time PCR was performed using the ABI PRISM 7000 Sequence Detection System (Applied Biosystems, Foster City, CA) according to the manufacturer's protocol. *HSP27* (Hs03044127_g1) and *HSF1* (Hs00232134_m1) primers and probes were designed by the manufacturer (TaqMan Gene expression assays; Applied Biosystems). Thermal cycling was performed using 40 cycles of 95°C for 15 seconds followed by 60°C for 1 min. Each experiment was done in triplicate, and the results were normalized to the *GAPDH* gene as an internal control.

### Western blotting analysis

Western blotting was performed as described previously [50]. Briefly, 1 × 10^5^ of ALDH^high^ cells and ALDH^low^ cells derived from HEC-1 cells, siRNA transfected HEC-1 cells and HSF1 plasmid transfected HEC-1 cells were lysed in 100 μl of SDS sample buffer. Anti-ALDH1 mouse monoclonal antibody (clone: 44/ALDH, BD Pharmingen) was used at 1000-times dilution. Anti-HSP27 rabbit polyclonal antibody (Abcam, Cambridge, UK), anti-HSF1 rabbit polyclonal antibody (Sigma-Aldrich, Saint Louis, MO), anti-HSF1 pSer320 rabbit polyclonal antibody (Abcam, Cambridge, UK), anti-HSF1 pSer326 rabbit polyclonal antibody (Abcam, Cambridge, UK), anti-HSF1 pSer230 rabbit polyclonal antibody (Santa cruz, Dallas, TX), anti-HSF1 pSer303 rabbit polyclonal antibody (Sigma-Aldrich, Saint Louis, MO) and anti-HSF1 pSer307 rabbit polyclonal antibody (Santa cruz, Dallas, TX) were used at 1000-times dilution. Anti-β-Actin mouse monoclonal antibody (Sigma-Aldrich, Saint Louis, MO) was used at 2000-times dilution. Anti-mouse IgG+IgM and anti-rabbit IgG and IgM second antibodies (KPL) were uses at 2000-times dilution. The membrane was visualized with Chemiluminescent HRP Substrate (Milipore Corporation, Bilerica, MA) according to the manufacturer's protocol, and pictures were taken by an Odyssey^®^ Fc Imaging System (LI-COR, Lincoln, NE).

### HSF-1 mRNA knockdown by siRNA

To knockdown HSP27 and HSF-1 genes, HSP27 siRNAs (Hs_HSPB1_2074(i), Hs_HSPB1_2076(ii)) and HSF-1 siRNAs (Hs_HSF1_7735(i), Hs_HSF1_7746(ii)) were purchased from Life Technologies. Transfection of siRNA duplexes using Lipofectamine™ RNAiMAX (Life Technologies) was performed according to the protocol of the manufacturer. Cells were transfected with siRNA 48 hours before analysis. Negative control siRNA (Stealth RNAi Negative Control; Life Technologies) was used.

### HSF1 and HSF1 mutants’ construction and overexpression

Full-length HSF1 cDNA was amplified from cDNA of HEC-1 cells with PCR using KOD-Plus DNA polymerase (Toyobo, Osaka, Japan). The PCR product was inserted into pIRES-puro3 expression vector (TaKaRa Bio, Kusatsu, JAPAN). HSF1 mutants (S230A, S230E, S303A, S303E, S307A, S307E, S320A, S320E, S326A and S326E) were constructed by PCR mutagenesis using mutation-specific primers, and the sequences were confirmed by DNA sequencing. HSF1 and HSF1 mutants coding plasmid was transfected into HEC-1 cells using Lipofectamine^®^ 2000 Transfection Reagent (Life Technologies) according to manufacturer's protocol. Tresnfected cells were cultured in puromycin (1 μg/ml) containing growth medium until stable transformants were established.

### Immunohistochemical staining

Immunohistochemical staining was performed with formalin-fixed, paraffin-embedded (FFPE) sections of tumors of human ovarian cancer cases and tumors derived from NOD/SCID mice as described previously [51]. Surgical specimens used for immunohistochemically staining were obtained from 122 patients with primary epithelial ovarian cancer who had been treated at Sapporo Medical University Hospital during the period from 2001 to 2011. Anti-ALDH1 monoclonal antibody (1:100, Sigma-Aldrich), rabbit anti-HSP27 polyclonal antibody (1:150, Abcam), rabbit anti-HSF1 polyclonal antibody (1:200, Sigma-Aldrich) and rabbit anti-HSF1 pSer326 polyclonal antibody (1:500, Abcam) were uses as 1^st^ antibodies.

### Statistical analysis

Statistical analysis, data fitting and graphics were performed by SPSS software package ver.19 (SPSS, Chicago, IL, USA). Data was shown as the mean *±* SD of at least 3 independent experiments and Chi-square test was used to assess the statistical significant difference (*p* < 0.05). Overall survival (OS), which was defined as interval from the date of first diagnosis to the date of death of disease progression were estimated using Kaplan-Meier method and compared with the log-rank test.

## SUPPLEMENTARY MATERIALS TABLES



## Abbreviations

CSC: cancer stem-like cell
CIC: cancer-initiating cell
ALDH1: aldehyde dehydrogenese 1
HSP: heat-shock protein
HSF1: heat-shock factor 1
