# Supplementary material for: Phosphorylation of HSF1 at serine 326 residue is related to the maintenance of gynecologic cancer stem cells through expression of HSP27
Source: Oncotarget. 2017 Mar 18;8(19):31540–53. doi: 10.18632/oncotarget.16361 (PMC5458228; doi:10.18632/oncotarget.16361)
Supplement: Supplementary file 1 [file oncotarget-08-31540-s001.pdf]

Phosphorylation of HSF1 at serine 326 residue is related to the maintenance of gynecologic cancer stem cells through expression of HSP27

Supplementary Materials

Supplementary Table 1: Primer sequences for RT-PCR

Gene Name		Primer sequence (5′→3′)	Product size (bp)
ALDH1A1	forward	TGTTAGCTGATGCCGACTTG	154
	reverse	TTCTTAGCCCGCTCAACACT	
SOX2	forward	GCCTGGGCGCCGAGTGGA	301
	reverse	GGGCGAGCCGTTCATGTAGGTCTG	
POU5F1	forward	TGGAGAAGGAGAAGCTGGAGCAAAA	186
	reverse	GGCAGATGGTCGTTTGGCTGAATA	
NANOG	forward	ATCCAGCTTGTCCCCAAAG	459
	reverse	ATTTCATTCGCTGGTTCTGG	
CD44	forward	AGGCTGGGAGCCAAATGAAG	83
	reverse	TGTGGTCAAAAGCCCGTGG	
ABCG2	forward	CACCTTATTGGCCTCAGGAA	206
	reverse	CCTGCTTGGAAGGCTCTATG	
HSP27	forward	GGAGTGGTCGCAGTGGTTAG	764
	reverse	GGACAGGGAGGAAACTT	
GAPDH	forward	ACCACAGTCCATGCCATCAC	452
	reverse	TCCACCACCCTGTTGCTGTA	

Supplementary Table 2: List of genes upregurated in HEC-1 ALDH1high cells

Gene symbol	Gene name	Accession no.	Expression ratio	
			Cy3/Cy5	Cy5/Cy3
TBX3	T-box 3(ulner mammary syndrome)	NM_016569	34.553	2.5109
HSD17B4	Hydroxysteroid (17-beta) dehydrogenase 4	NM_000414	26.865	2.197
MAPK13	Mitogen-activated protein kinase 13	NM_002754	22.685	2.155
UBQLN2	Ubiquilin 2	NM_0013444	10.524	2.022
TAIP-2	TGF-beta induced apoptosis protein 2	NM_ 024969	10.212	2.879
BCL7B	B-cell CLL/lymphoma 7B	NM_001707	7.732	2.081
IL1B	Interleukin1, beta	NM_ 000576	7.183	2.87
SEPW1	SelenoproteinW 1	NM_003009	4.453	2.159
HSPB1	Heat shock 27kDa protein 1	NM_001540	3.547	2.718
PTTG1IP	Pituitary tumor-transforming1, interacting protein	NM_004339	2.599	2.302
CAPN1	Calpain 1, large subunit	NM_005186	2.487	2.073
MPL	Myeloproliferative leukemia virus oncogene	NM_ 005373	2.296	2.175

Genes showing the ratio more than 2.0, which were reproductible in two experiment, were listed.

Supplementary Table 3: Summary of patients clinicopathological status

Characteristic		pHSF expression				Total
		0 (N = 41)	1 (N = 40)	2 (N = 41)	P	(N = 122)
Age	Mean ± SD	11	10	11	0.91	55.1 ± 10.2
	Range	55	55	55		29–81
Parity (%)	0	10	14	15	0.72	39 (32)
	1	12	7	7		26 (21.3)
	2	14	14	12		40 (32.8)
	3	4	4	7		15 (12.3)
	4	1	1	0		2 (1.64)
Histological subtype (%)	Serous	16	20	25	0.14	61 (50)
	Clearcell	15	12	10	0.49	37 (30.3)
	Endometrioid	6	7	5	0.80	18 (14.8)
	Mucinous	4	1	1	0.21	6 (4.92)
FIGO Stage No. (%)	I	22	11	9	0.001**	42 (34.4)
	II	5	0	1		6 (4.92)
	III	13	27	26		66 (54.1)
	IV	1	2	5		8 (6.56)
Peritoneal dissemination (%)		15	27	29	0.003**	71 (58.2)
Lymph node metastasis (%)		9	9	18	0.04*	36 (30)
Optimal debulking surgery (%)		32	21	18	0.008**	72 (59)
Platinum resistant (%)		7	23	20	< 0.001	50 (41)
